# Preparation of Astaxanthin Nanodispersions Using Gelatin-Based Stabilizer Systems

**DOI:** 10.3390/molecules190914257

**Published:** 2014-09-10

**Authors:** Navideh Anarjan, Imededdine Arbi Nehdi, Hassen Mohamed Sbihi, Saud Ibrahim Al-Resayes, Hoda Jafarizadeh Malmiri, Chin Ping Tan

**Affiliations:** 1 Department of Engineering, College of Chemical Engineering, Tabriz Branch, Islamic Azad University, Tabriz 51368, Iran; E-Mail: anarjan@iauasrb.ac.ir; 2 Chemistry Department, College of Science, King Saud University, Riyadh 1145, Saudi Arabia; E-Mails: inahdi@ksu.edu.sa (I.A.N.); hmsbihi@ksu.edu.sa (H.M.S.); sresayes@ksu.edu.sa (S.I.A.-R.); 3 Department of Chemical Engineering, Faculty of Food Engineering, Sahand University of Technology, Tabriz 51368, Iran; E-Mail: h_jafarizadeh@sut.ac.ir; 4 Department of Food Technology, Faculty of Food Science and Technology, Putra University, 43400 UPM Serdang, Selangor, Malaysia

**Keywords:** astaxanthin nanodispersions, emulsifying mixture, emulsification-evaporation

## Abstract

The incorporation of lipophilic nutrients, such as astaxanthin (a fat soluble carotenoid) in nanodispersion systems can either increase the water solubility, stability and bioavailability or widen their applications in aqueous food and pharmaceutical formulations. In this research, gelatin and its combinations with sucrose oleate as a small molecular emulsifier, sodium caseinate (SC) as a protein and gum Arabic as a polysaccharide were used as stabilizer systems in the formation of astaxanthin nanodispersions via an emulsification-evaporation process. The results indicated that the addition of SC to gelatin in the stabilizer system could increase the chemical stability of astaxanthin nanodispersions significantly, while using a mixture of gelatin and sucrose oleate as a stabilizer led to production of nanodispersions with the smallest particle size (121.4 ± 8.6 nm). It was also shown that a combination of gelatin and gum Arabic could produce optimal astaxanthin nanodispersions in terms of physical stability (minimum polydispersity index (PDI) and maximum zeta-potential). This study demonstrated that the mixture of surface active compounds showed higher emulsifying and stabilizing functionality compared to using them individually in the preparation of astaxanthin nanodispersions.

## 1. Introduction

Astaxanthin is a fat-soluble pigment belonging to the xanthophyll family and is widely used in food and pharmaceutical applications due to its strong antioxidant activity. Enormous health benefits, such as cardiovascular disease prevention, immune system boosting, bioactivity against *Helicobacter pylori*, and cataract prevention have been associated with astaxanthin consumption [1,2,3]. However, as for other carotenoids, the insolubility of astaxanthin in water results in low bioavailability and seriously limits its applications in aqueous-based systems [4].

Nanotechnology has provided solutions for improving the water solubility and bioavailability of these bioactive lipophilic compounds. Emulsification-evaporation, emulsification-freeze-drying, emulsification-diffusion, solvent displacement, solvent evaporation within porous polymers and precipitation methods are some of the various methods that have been developed for the preparation of water-based functional lipid compound nanodispersions [5,6,7,8]. Emulsification-evaporation is one of the most favored techniques for preparing carotenoid nanodispersions. In this method, the organic phase, a lipophilic active compound dissolved in a water-immiscible solvent, is emulsified into an aqueous phase containing an emulsifier by appropriate high-pressure or high-shear emulsifying techniques, followed by converting the emulsion into nanodispersion by evaporating the solvent. The precipitation or crystallization of the active compound occurs in the O/W emulsion droplets during evaporation when the solubility limit is reached. The removal of the solvent from the emulsion droplets decreases the particle size to the nano-size range [5].

The bioactive-compound particles in nanodispersions are stabilized by an emulsifier or a mixture of emulsifiers. The emulsifiers are surface-active molecules that should be adsorbed at the interface of two phases, decrease the interfacial tension and prevent the aggregation and re-coagulation of particles [9,10,11]. Proteins are large, complex amphiphilic molecules with combinations of polar and non-polar regions that can be used to prepare food emulsions [12]. A major potential advantage of proteins as emulsifiers in foods is their ability to protect polyunsaturated lipids from oxidation [13,14].

Gelatin is a relatively high molecular weight protein derived from animal collagen [14]. It is prepared by hydrolyzing collagen by boiling in the presence of either acid (Type A gelatin) or alkaline (Type B gelatin). The relatively high isoelectric point (pI ≥ 7.0) of Type A gelatin means that it should be possible to create emulsions/dispersions that have a positive charge over a wider range of pH values. Consequently, Type A gelatin may be suitable for creating oil-in-water food emulsions with high oxidative stability, as it could repel iron ions from oil droplet surfaces over most of the pH range typically found in foods [15].

Therefore, the purpose of this study was to prepare astaxanthin nanodispersions using gelatin alone and in combination with other surface-active molecules (sucrose oleate, SC and gum Arabic) and to evaluate their effects on the formation and characteristics of the resulting nanodispersions. All three mentioned surface active compounds have proved to have the best emulsifying properties among other small molecular emulsifiers, proteins and polysaccharides, respectively [16].

## 2. Results and Discussion

**Figure 1:**
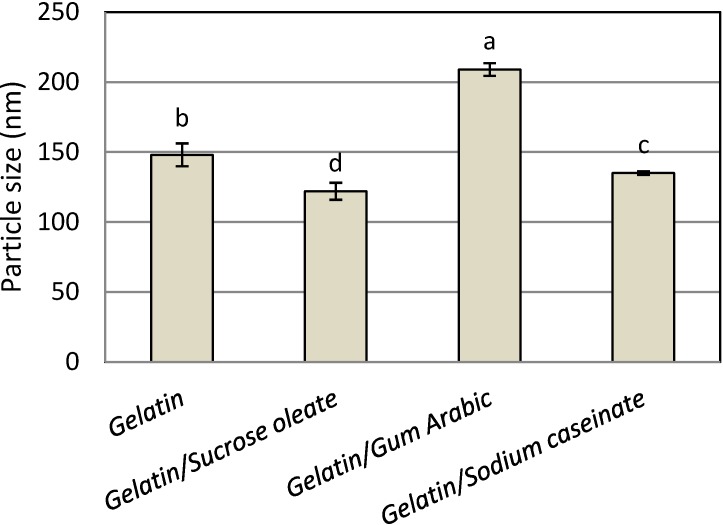
Mean particle size of freshly prepared astaxanthin nanodispersions stabilized with different gelatin based emulsifying systems.

*Notes*: Values are means ± standard deviations (*n* = 4); ^a−d^ Different letters indicate statistically significant differences between treatments (*p* < 0.05).

Figure 1 shows the mean particle size of the astaxanthin nanodispersions prepared with different emulsifier (stabilizer) systems. The mean nanometer size range (122–209 nm) of the astaxanthin particles confirmed the suitability of all emulsifying and stabilizing systems in the preparation of astaxanthin nanodispersions through the emulsification-evaporation technique. The use of gelatin alone as a one-component stabilizer led to the production of astaxanthin nanodispersions with a mean particle size of 148 nm. The astaxanthin nanodispersions prepared with a mixture of gelatin and sucrose oleate had the smallest particle size (122 ± 6.1 nm). The interfacial behavior of the different emulsifier systems may contribute to the differences in droplet sizes [17]. Sucrose oleate is a small molecular emulsifier with much lower surface tension than water, showing low interfacial tension when mixed with the organic phase, which favors the formation of small droplets [16,17,18], therefore, the combination of this compound with gelatin produced nanodispersions with smaller particle sizes than using gelatin individually or in combination with SC and gum Arabic. In contrast, the large molecular structures of SC and gum Arabic would prevent close packing of the points of contact with the interface, resulting in relatively high interfacial tension [17,18], and these molecules also have low adsorption kinetics [17,19], so their inability to efficiently and quickly stabilize the newly produced droplets can lead to re-coalescence of the droplets [17,18]. For the blend of gelatin with SC and gum Arabic, nanodispersions with smaller particle sizes were produced from SC (135 ± 1.2 nm) than from gum Arabic (209 ± 4.5 nm). This result could be explained by a possible conformation change of SC during and after homogenization, as the protein chain might unfold during adsorption, resulting in the exposure of more hydrophobic groups, which would facilitate emulsification [17,20]. It seems that the possible conformation changes for gelatin were considerably less than for the mixture of gelatin and SC. Therefore, it can be concluded that among all stabilizer systems, combination of gelatin and sucrose oleate was the best choice in terms of mean particle size of products.

**Figure 2:**
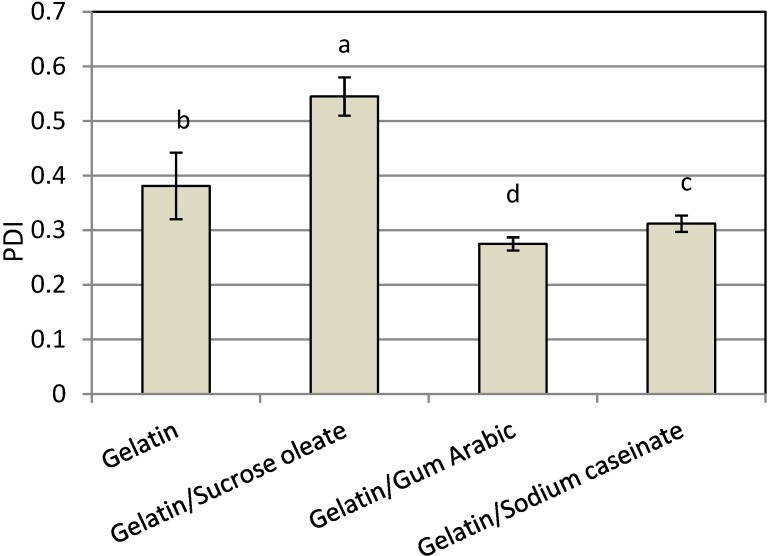
Mean PDI of freshly prepared astaxanthin nanodispersions stabilized with different gelatin-based emulsifying systems.

*Notes*: Values are means ± standard deviations (*n* = 4); ^a−d^ Different letters indicate statistically significant differences between treatments (*p* < 0.05).

**Figure 3:**
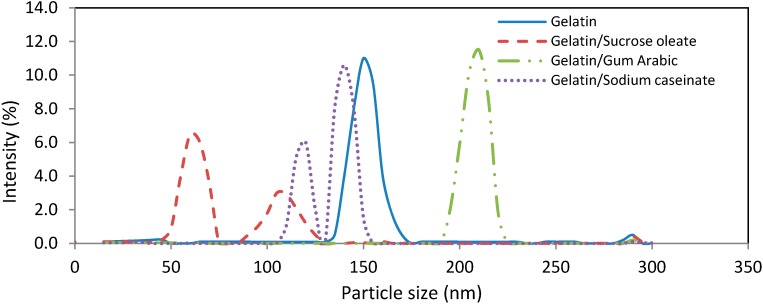
Particle size distribution of freshly prepared astaxanthin nanodispersions stabilized using different gelatin-based emulsifying systems.

Among the nanodispersions produced, the result of using a combination of gelatin with gum Arabic had the smallest polydispersity index (PDI, Figure 2). The PDI was calculated as the best fit between the measured scattered pattern and the pattern predicted by light-scattering theory [21]. The particle size distribution of nanodispersions produced by gelatin (0.381 ± 0.074) and mixtures of gelatin and gum Arabic (0.275 ± 0.012) were mono-modal, while the size distributions produced by a combination of gelatin with SC (0.312 ± 0.025) or sucrose oleate (0.454 ± 0.040) was bimodal. The systems with narrow size distributions exhibited high physical stability due to their low affinity for all destabilization phenomena, especially Oswald ripening, during the storage [17,22]. Consequently, the mixture of gelatin and gum Arabic was considered as the best stabilizer system in terms of the PDI of produced nanodispersions (0.275 ± 0.062). The particle size distribution of samples was shown in Figure 3.

**Figure 4:**
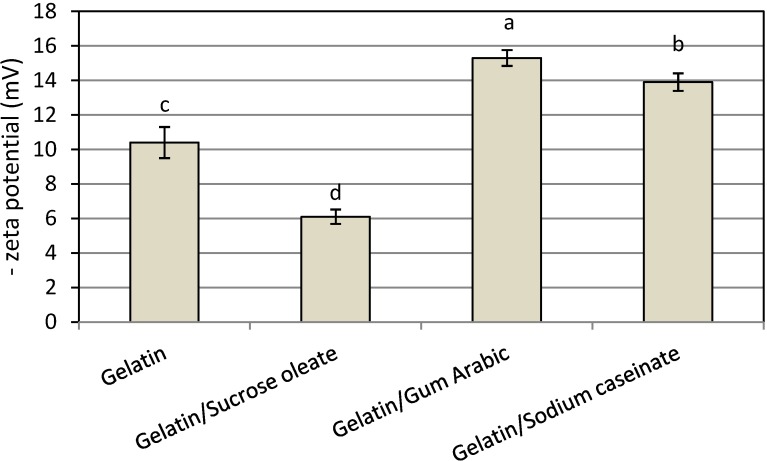
Mean zeta potential of freshly prepared astaxanthin nanodispersions stabilized with different gelatin based emulsifying systems.

*Notes*: Values are means ± standard deviations (*n* = 4); ^a−d^ Different letters indicate statistically significant differences between treatments (*p* < 0.05).

In this work, all nanodispersions produced showed a negative surface charge (zeta potential), and the mixture of gelatin and gum Arabic produced the nanodispersion with the highest net surface charge (15.3 ± 0.56 mV, Figure 4). The adsorption of OH species from the aqueous phase or cationic impurities from astaxanthin onto the interface could be responsible for the observed negative surface charges [16,17,23]. Therefore, the combination of gelatin and gum Arabic was the most desirable stabilizer system from either PDI or net zeta potential value, and consequently, total physical stability of the prepared astanxanthin nanodispersion.

**Figure 5:**
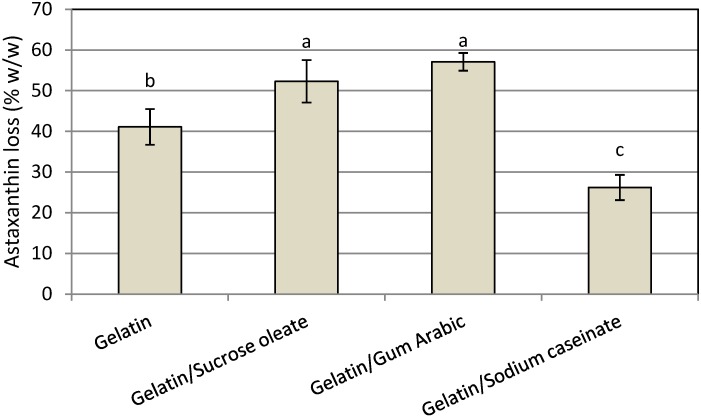
Mean astaxanthin loss (% w/w) of freshly prepared astaxanthin nanodispersions stabilized with different gelatin based emulsifying systems.

*Notes*: Values are means ± standard deviations (*n* = 4); ^a−c^ Different letters indicate statistically significant differences between treatments (*p* < 0.05).

**Figure 6:**
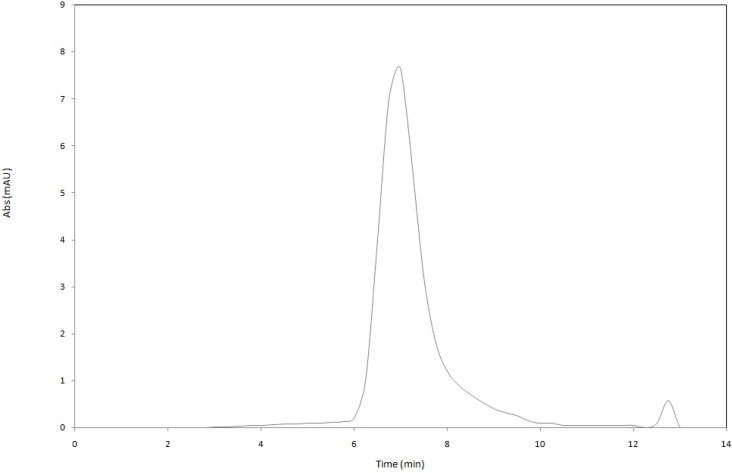
Representative HPLC chromatogram of extracted astaxanthin from the gelatin/SC stabilized nanodispersion.

The chemical stability of functional lipid nanodispersions is one of the most important product quality issues. The HPLC results indicated that astaxanthin degradation occurred in all prepared nanodispersions due to the high sensitivity of the unsaturated astaxanthin chemical structure to thermal and oxidative stresses during the processing steps (Figure 5). The degradation of astaxanthin in nanodispersions during processing might be seriously accelerated by the large surface area of the prepared astaxanthin particles as a result of size reduction to the nanometer range as well as by the possible creation of free radicals during high-pressure homogenization process [8,9,17,24]. In this research, the chemical stability of astaxanthin nanodispersions was strongly influenced by the emulsifier systems used. The mixture of gelatin with SC showed the least degradation of astaxanthin (26.2% ± 3.1%) during the processing steps because of the antioxidant activity of SC due to the presence of cysteinyl residues, disulfide bonds and thiol functional groups on its structure, which can prevent lipid oxidation by scavenging free radicals [9,18]. Astaxanthin was less stable in nanodispersions prepared using the mixture of gelatin with either sucrose oleate (52.3% ± 5.22%) or gum Arabic (57.1% ± 2.18%). Thus, for better protection of astaxanthin in nanodispersed form, a mixture of gelatin and SC as stabilizer system was a good option. The HPLC chromatogram of extracted astaxanthin from the gelatin/SC stabilized nanodispersions is shown in Figure 6.

## 3. Experimental Section

### 3.1. Materials

Astaxanthin (>90%) was purchased from Kailu Ever Brilliance Biotechnology Co., Ltd. (Beijing, China). Sucrose oleate (OWA-1570) were donated by Mitsubishi Food Co. (Tokyo, Japan). Sodium caseinate (SC) and gum Arabic were provided by Merck Co. (Darmstadt, Germany). Gelatin type A (polyoxythylene sorbitan monolaurate) were bought from Sigma Aldrich (Steinheim, Germany). Sodium azide, analytical and HPLC-grade dichloromethane, methanol and acetonitrile were provided by Fisher scientific (Leicestershire, UK). All chemicals were used without future purification.

### 3.2. Preparation of Astaxanthin Nanodispersions

Gelatin or a mixture of gelatin with sucrose oleate/SC/gum Arabic (1:1 w/w) were dissolved in deionized water (at 20 °C), containing 0.02 wt % sodium azide at a concentration of 1% w/w and thoroughly mixed by magnetic stirring for 4 h. The organic phase, consisting of dissolved astaxanthin in dichloromethane (1% w/w), was added to the aqueous phase, and coarse emulsions were formed by two-phase homogenization using a conventional homogenizer (Silverson, Buckinghamshire, UK) at 5000 rpm for 5 min. By placing the system in a high-pressure homogenizer (APV, Crawley, UK) at 50 MPa twice, fine emulsions (nanoemulsion) were produced. The organic to aqueous phase ratio was set at 1:9. The sample volume of 300 mL was used for each sample.The dichloromethane was then removed from the system by rotary evaporation (Eyela NE-1001, Tokyo Rikakikai Co. Ltd., Tokyo, Japan) at 250 Pa and 47 °C and 100 rpm to convert the astaxanthin nanoemulsions into astaxanthin nanodispersions.

### 3.3. Analytical Methods

Measurement of the mean particle size, PDI, zeta potential, mobility and conductivity of produced astaxanthin particles were conducted by using Zetasizer Nano ZS (Malvern Instruments Ltd., Worcestershire, UK). The absorbance of the nanodispersion particles was set at 0.3. To avoid multiple scattering effects, the dispersions were diluted with deionized water prior to analysis and then directly placed into the module immediately after preparation. Measurements were performed at 25 °C in triplicate [24].

The measurement of astaxanthin concentration in prepared astaxanthin nanodispersions was performed using an Agilent liquid chromatography system (Agilent Technologies 1200 Series, Waldbroon, Germany), equipped with a Diode Array Detector G13150, a Nova-Pak^®^ C18 (3.9 × 300 mm) Waters HPLC column and an isocratic mobile phase consisting of 85% methanol, 5% dichloromethane, 5% acetonitrile and 5% water. The absorption spectra were in the range of 250–700 nm intervals, and detection was performed at 480 nm. The calibration of peak area *versus* astaxanthin concentration was linear in the measurement concentrations [25].

### 3.4. Statistical Analysis

The physicochemical characteristics of astaxanthin nanodispersions were subjected to one-way analysis of variance using the Minitab v. 14 statistical package (Minitab Inc., University Park, PA, USA). All experiments and measurements were performed in duplicate and Tukey’s multiple range tests were used to determine significant differences (*p* *<* 0*.*05) between the responses.

## 4. Conclusions

Gelatin itself could successfully produce colloidal astaxanthin particles by the emulsification-evaporation method at nanometer sizes. The physicochemical characteristics of the produced nanodispersions was less desirable compared to, for instance, sucrose oleate- or SC-stabilized nanodispersions, which could be explained by insufficient coverage of all particle surfaces created within the homogenizer, relatively low surface activity, the slow adsorption kinetics of the gelatin within the homogenizer and its disability to completely cover the freshly formed droplet surfaces and prevent their re-coalescence [17]. However, the combination of gelatin with other active compound molecules improved the physicochemical characteristics of obtained nanodispersions. Generally, suitable combinations of emulsifiers can enhance desired emulsion/dispersion properties compared to using them individually, due to the formation of intermolecular complexes at interfaces [17,26].
